# Incorporating an equity perspective in systematic reviews of interventions: potential methodological approaches

**DOI:** 10.1136/jech-2025-224306

**Published:** 2025-12-19

**Authors:** Mhairi Campbell, G.J. Melendez-Torres, Vivian Welch, Jennifer Petkovic, Ffion Curtis, S Vittal Katikireddi

**Affiliations:** 1 School of Health and Wellbeing, University of Glasgow, Glasgow, UK; 2 University of Exeter, Exeter, UK; 3 Bruyere Health Research Institute, University of Ottawa, Ottawa, Ontario, Canada; 4 University of Liverpool, Liverpool, UK

**Keywords:** Health inequalities, PUBLIC HEALTH, SYSTEMATIC REVIEW, RESEARCH DESIGN

## Abstract

Health inequities are unnecessary, avoidable and unjust differences in health across social groups. Addressing them is a priority for governments and health systems worldwide, requiring not only specific interventions targeting inequity but also embedding equity across all decision-making. Systematic reviews of interventions underpin health decision-making and could, therefore, be a key mechanism to address inequities, but most reviews are limited in their approach to considering equity and often only conclude data for subgroup analyses are unavailable. While some guidance is available, it largely focuses on reviews of interventions specifically seeking to reduce inequities and is published in disparate literature. We describe approaches to incorporate an equity perspective relevant to all systematic reviews of interventions, even when equity is not the primary review focus.

Consideration of equity may be needed at all stages of the review process. Planning the review involves examining theory, using logic models, involving relevant people and organisations, and considering if additional sources of evidence are needed. Investigating the data requires examining the external validity of primary studies, including who was involved in the primary studies, and the reach of interventions. The synthesis process includes selecting appropriate analysis, considering the implications of reporting absolute or relative equity effects of the intervention, exploring and understanding mechanisms and assessing certainty of the evidence in relation to equity. Interpreting results involves linking theory with evidence and discussing implications and limitations. We hope this article helps review authors make best use of the available evidence to incorporate equity into systematic reviews.

**WHAT IS ALREADY KNOWN ON THIS TOPIC**

- Systematic reviews are crucial for informing policy and practice, but they often do not examine equity and when they do, are limited to assessing differential intervention effectiveness.

**WHAT THIS STUDY ADDS**

- Approaches to incorporating equity throughout the systematic review process are described, from initial conceptualisation of the review through to interpretation of the review findings.

**HOW THIS STUDY MIGHT AFFECT RESEARCH, PRACTICE OR POLICY**

- Considering equity in all systematic reviews increases the potential for practitioners and policymakers to take action to reduce inequities and highlight evidence gaps to be addressed in future research.

## Introduction

 Health inequity, that is, unnecessary, avoidable and unjust differences in health across social groups,^1 2^ has been identified as a priority challenge by the WHO.^3^ While health inequalities are differences in health between populations, health inequity is when these differences are judged to be unfair.^1^ Often the two terms are used interchangeably and, in the USA, the term ‘health disparities’ is frequently used.^4^ Systematic reviews of interventions are a key resource for health decision makers. Therefore, it is important for them to examine whether, and how, outcomes differ according to equity-deserving population characteristics, that is, features people have that can lead to unjust differences in health usually through discrimination from organisations or in society. For example, smoking cessation media campaigns are often less effective in equity-deserving populations than for advantaged population groups.^5^ However, current reviews arguably take a limited approach to equity. While targeted interventions can be important for health inequalities, it is often equally important to examine broader policies and interventions not primarily focused on reducing health inequity. For example, in a review of interventions to address youth homelessness, differential treatment effects were found based on gender and ethnicity.^6^ In addition, many interventions can increase inequities rather than reduce them^7^; therefore, reviews should consider equity in some way.

While some systematic reviews focus on specific equity-deserving populations (see box 1 and online supplemental file for terminology), many reviews not focusing on specific populations examine interventions, which may have important health equity implications.^8^ When such reviews consider equity, they often seek to establish differential effects across population subgroups. However, systematic reviews frequently find a lack of relevant data. New approaches are, therefore, needed to maximise the use of available evidence to inform decision-making around health equity. There are resources to help conduct equity-focused systematic reviews^8^,^10^ (see box 2 and online supplemental file). However, there may be further considerations, particularly when equity is not the main purpose of the review, and existing resources can be difficult to navigate to understand relevant concepts and relate the guidance to the specifics of the user’s review. We provide key points, with examples, to consider (table 1 and online supplemental file) for considering equity in all systematic reviews of interventions, across different review stages.

Box 1 **Terminology related to health equity**

**Health inequity**: unnecessary, avoidable and unjust differences in health, when the differences in health are judged to be unfair.^A1^ Sometimes the term ‘health inequality’ is also used to mean health inequity, and in the USA, ‘health disparities’ is frequently used.

**Equity-deserving groups, equity-deserving populations**: we use these terms to acknowledge that structural barriers create inequity and people impacted by structural and societal inequalities deserve equity. The term ‘equity seeking’ is also used, particularly in North America.^A1^ Discussions continue to find suitable non-stigmatising terminology.^A2^

**Interest holder**: we use this term in place of stakeholder, which has negative historical uses, for example, someone who marked land taken from Indigenous peoples. Interest holders are people and organisations whose interests should be considered for health research. Interest holders include people with lived experience, healthcare providers, practitioners, policymakers, funders of health services and health research, researchers and the public.^A3^

PROGRESS-plus categories*: place of residence, race/ethnicity/culture/language, occupation, gender/sex, religion, education, socioeconomic status and social capital.^A1^

Plus categories*: ‘personal characteristics that attract discrimination (eg, age and disability). Features of relationships (eg, smoking parents, excluded from school). Time-dependant relationships (eg, leaving the hospital, respite care and other instances when a person may be temporarily at a disadvantage).’ O’Neill *et al*^A1^ (page 61).

EQUALS MAP categories*: ethnicity, race and language; qualifications, income, wealth, education, class and employment; underprivileged/socioeconomically disadvantaged areas; age; sexual identity; sex and gender; multiple disadvantage; additional specific groups experiencing significant disadvantage; and physical and mental disability.^A7^

References available in online supplemental file 1.

*It is unlikely that all categories will be relevant in the review.

Box 2 **Guidance for researchers incorporating equity into systematic reviews**

**Chapter 16 Equity and specific populations:** Cochrane Handbook chapter on incorporating equity into systematic reviews of interventions.^B1^

**PRISMA-E 2012:** explanation and elaboration for extending the preferred reporting items for systematic reviews and meta-analyses (PRISMA) reporting guidance for reporting equity-focused systematic reviews.^B2^

**Campbell and Cochrane equity methods group webpage:** website of resources for planning, conducting and reporting equity-relevant systematic reviews.^B3^

**Grading Recommendations Assessment and Development Evidence (GRADE) equity guidelines:** guidance on considering health equity in GRADE guideline development.^B4-S7^

**Chapter 20 Economic evidence:** Cochrane Handbook chapter on incorporating economic evidence into systematic reviews of interventions.^B8^

**Guidance that can be useful for reviews PROGRESS Plus:** a guide to identify characteristics that relate to health opportunities and outcomes.^B9^

**WHO-INTEGRATE (INTEGRATe EVIDENCE) framework:** an evidence to decision framework providing a structured process for the inclusion of WHO norms and values criteria in guideline development and health decision-making.^B10^

**Sex and gender equity in research (SAGER) guideline:** a checklist for comprehensively reporting sex and gender information in studies.^B11^

**PRO-EDI participant characteristics table:** table for reporting participant information from studies to facilitate consideration of equity and inclusion.^B12^

**Health equity impact assessment (HEIA) tool:** a decision support tool to examine how a policy or programme affects health equity in different population groups.^B13^

**Health equity assessment toolkit(HEAT—WHO):** software application to facilitate assessment and communication of data about health inequalities.^B14^

References available in online supplemental file 1.

**Table 1:** Summary of equity considerations with examples

Component of the review	Considerations	Examples of equity consideration*
Focus of review	Consider what the relevant equity questions are for the review.	Provided justification from published studies for the equity-related subgroup meta-analyses (sex, age, ethnicity and place of residence) (Review examining use of systemic corticosteroids treatment for COVID-19).^T1^
Protocol/planning the review	Examine how the intervention mechanisms operate across equity groups, ensuring the logic model and theory of change show how the intervention is expected to impact on equity-deserving populations, including harms and benefits. Consider whether a systems map would be useful. Consider how to incorporate involvement with interest holders, including people with relevant lived experience. Identify PROGRESS-plus or EQUALS MAP populations relevant to the intervention under investigation, justifying why equity matters and inclusion of specific equity-deserving populations in theory of change and/or logic model. Define axes of inequalities—identify and give rationale for which PROGRESS-plus characteristics (or their intersection) are likely to impact on equity. Define the scales used to assess inequity between populations, implications of using different scales and what different scales convey.	Included equity-deserving characteristics in a logic model to understand the mechanisms of action of the intervention (Review of interventions reducing harmful lifestyles in street‐connected children).^T2^ Included lived experience of young people to inform understanding of definitions and relevance of findings in the review (Review of religiosity and spirituality for depression and anxiety in young people).^T3^ Used harvest plots to synthesise evidence for different equity-deserving groups, including gender, place of residence, social capital, ethnicity, age and disability (Review examining equity in primary-care-based physical activity interventions).^T4^
Representation—in included studies	Examine what populations are included in the studies included in the review, informed by RE-AIM. Identify appropriate data for analysis. Identify evidence gaps.	A review of interventions to reduce disciplinary school exclusion examined the studies included in the review to determine whether data were available for different ethnicities and socioeconomic status.^T5^
Representation—reach	Consider the intervention design and implementation to understand and examine what populations the interventions aimed to reach and which populations were actually reached in studies included in the review.	Examined the reach, that is, the intended and actual recipients of smoking cessation campaigns to understand what evidence was available about equity-deserving groups.^T6^
Mechanisms in the theory of change	Revisit the theory of change for the review based on the results of included studies; a metaframework may be of use. Consider the intervention design and implementation to explore how intervention design and implementation impact equity-deserving populations, for both intended and unintended consequences. Explore mechanistic evidence. Draw on qualitative evidence, process evaluation or relevant evidence beyond the included studies to understand how the intervention impacts equity.	Logic model included equity characteristics mediators of the effect of the interventions. The PROGRESS-Plus framework was used to examine inequity categories in a review of social capital interventions for older people.^T7^ Included evidence from qualitative studies to better understand barriers and facilitators to interventions for common mental health disorders (Review of primary-care interventions to reduce inequalities in common mental health disorders).^T8^
Analysis of effectiveness	Consider information different scales provide, what comparisons are appropriate. Consider subgroup analysis, using tools, such as harvest plots or individual participant data meta-analysis if appropriate. Ensure that subgroup analyses are justified and preferably preplanned and limited in number. Subgroup analysis may involve using tables or figures, such as harvest plots.	Individual participant data meta-analysis, using individual data from multiple studies rather than aggregate data to investigate effects for characteristics, including low income, low educational attainment and ethnic minority status (Review of parenting interventions for child conduct problems).^T9^ Conducted subgroup meta-analysis to examine whether there were differences in maternal and child health indicators according to income, education and place of residence.^T10^ Assessed equity in cancer prevention from human papillomavirus vaccination by examining both absolute and relative measures of inequity.^T11^ Assessed relative and absolute risk measures for each effect estimate (Review of mobile interventions for common mental disorders in pregnant and postpartum women).^T12^ Investigated whether the implementation of guidance resulted in different results for different equity-deserving populations (Review of inequalities in the identification and management of common mental disorders in the perinatal period). ^T13^
Economic evidence	Be aware of the limitations of cost-effectiveness analysis in relation to equity and the potential of methods, such as extended effectiveness analysis and distributional cost-effectiveness analysis to incorporate health equity judgements.	Distributional cost-effectiveness analysis used to examine the impact of screening on equity-deserving groups (Review of screening for familial hypercholesterolaemia). ^T14^
Interpreting results	Consider strength of evidence (applying GRADE equity as appropriate) and statistical results in relation to evidence from the theory of change and any additional sources.	Examined the extent to which equity is considered in guidelines, including assessing the certainty of the evidence using an equity perspective using GRADE equity (Review of equity in traumatic brain injuries clinical practice guidelines for the criminal justice system).^T15^

* References in online supplementary file.

GRADE, Grading Recommendations Assessment and Development Evidence.

This article aims to assist systematic reviewers to incorporate equity in their reviews, especially when the intervention being studied is not primarily introduced to address equity. The interventions may directly impact health or indirectly via determinants of health. The guidance is derived from a broad range of literature known to the authors who have experience of conducting primary research and evidence synthesis in the field of inequity in population health. This was supplemented with a purposive scan of the literature for relevant information and examples. We describe different aspects of equity and identify several stages of the review in which equity considerations may be important: deciding the review focus, planning the review, and when managing the data, conducting analysis and interpreting results. While not all these approaches will be required for every review, we hope this article provides reviewers with a suite of options from which they can identify feasible strategies to better integrate equity into reviews.

## Equity focus of the review

Prior to planning the review, it is helpful to consider what the review aims to investigate in relation to equity. Considering different mechanisms of the intervention components and equity-deserving populations is likely to be useful. There are multiple ways equity might be examined.

- *Mechanisms between groups*: mechanisms may differentially operate across equity-deserving groups. Examining how mechanisms operate across equity-deserving groups can be useful, including identifying and understanding the relationships among intervention components, equity-deserving groups and local context.
- *Representation*: studies may under-represent groups. The review may examine representation of equity-deserving populations in primary studies.
- *Intervention design and implementation*: interventions may be less likely to reach or be less acceptable across groups. A consideration may be how interventions can be tailored for different groups of people or how implementation of the interventions impacts and is impacted by equity factors. Another consideration may be how the design or implementation of an intervention impacts how the intervention is experienced by people with different equity characteristics.
- *Effectiveness*: effectiveness may differ across groups. The review may investigate whether there is differential effectiveness of the interventions in relation to equity factors.

Considering different aspects of equity, and what equity focus the review should have, will inform the review questions and how the review will be planned and conducted. It may be more useful for the review to investigate mechanisms of the intervention rather than, or in addition to, obtaining an overall effect size estimate.^11^ Reviews examining equity may be more complex and exploring how interventions work for different populations may provide valuable information.^12^ Input from interest holders at this stage, and throughout the review, is valuable for increasing the relevance of the review,^13^ with guidance available that prompts review authors to consider who, when and how interest holders will be involved.^14^

## Considering equity when planning the review

When planning the review, the protocol should define the inequities relevant to the intervention.^8 9^ This involves defining mechanisms of the intervention effects. This includes identifying populations that may be impacted differently and unfairly and understanding how the intervention components and implementation may relate to inequities that are created and perpetuated by underlying social structures and political, economic and legal systems.^15^ This may be considering the aspects of inequity that are important to understand in the review, for example, which equity-deserving populations experience disproportionate disease burden that are likely to be impacted by the intervention and how the inequitable outcomes occur. The PROGRESS Plus^16^ and EQUALS MAP^17^ frameworks are tools to help identify inequity factors, which outline different characteristics of disadvantage (box 2). Not all categories will be relevant for a review. Deciding which inequity factors to examine can be informed by evidence from relevant theories, pre-existing evidence and knowledge from people with lived experience, service providers and other relevant interest holders.^14^ Deciding which outcomes are relevant will depend on the contexts of equity-deserving populations in the review.^9^ It can be the case that there are several interconnected factors relevant to individual identities that interact through structural oppressions and privileges to influence health, this is known as intersectionality.^18 19^ Currently, methods for incorporating intersectionality into systematic reviews require further methodological development. However, we suggest considering which axes of inequity and their intersections are most relevant to the review to mitigate the risks of conducting too many or too fine-grained subgroup analyses and consequently overinterpreting potentially spurious findings. Understanding which axes of inequities are relevant can include acknowledging knowledge gaps that result from lack of intersectionality data, as well as engagement with interested communities.

When investigating equity within systematic reviews, logic models, theories of change and programme theories may be useful both before and during the review process.^9^ System mapping may be a useful alternative,^20^ such as creating a causal loop diagram of the intervention theory of change, mapping how variables interact and including feedback loops that illustrate nonlinear and circular interactions between variables.^21^ Even when equity is not the primary focus of a review, it is important to consider equity when developing the theory of change. Developing the logic model and theory of change may have implications for the intended purpose of the review, that is, what the review will investigate. Therefore, when the review question is being formulated, review authors decide whether relevant equity-deserving populations are specified in the primary review question, or whether it is more appropriate for equity to be explored as a secondary question.^9^

The type of relevant evidence in a review may be entirely qualitative or quantitative, or some combination of both. Restricting inclusion to randomised controlled trials, which often under-represent disadvantaged populations, could miss data for equity-deserving populations.^9^ Pragmatic or real-world non-randomised studies may be more appropriate for providing data on equity-deserving populations. For example, randomised controlled trials often do not examine the intervention in relation to the specific context of the intervention, that is, how it was implemented and received.^22^ Qualitative approaches can be valuable for investigating equity. Therefore, when relevant, the review design and protocol of a review of primarily quantitative evidence may need to include steps to source supplementary qualitative evidence.^23^ Information from interest holders, including people with lived experience or community representatives, is important when planning the review process to ensure that relevant questions are addressed, relevant data are collected and to determine other stages of the review where input from interest holders will be required.^14^

## Representation of equity-deserving populations in the included studies

In reviews where inequity is not the primary focus, it is possible that included studies under-represent equity-deserving groups. While an intervention may have been implemented universally, there may have been restrictions on the sampling strategy of studies investigating that intervention. For example, in large surveys of the ‘general’ population, sometimes certain groups are not included, for example, Indigenous populations^24^ or non-household populations.^25^ Therefore, it is necessary to examine which equity-deserving populations were in the included studies, to what extent and whether there were any groups excluded in the studies.^10^ Not including sections of the population in studies has implications: groups excluded from some studies may be under-represented in the synthesis of those studies, the acceptability, feasibility and effects of the intervention may not be the same for the excluded groups as that of the included population and the exclusion of some groups could undermine trust in the evidence and intervention acceptability.

Understanding and demonstrating which relevant equity-deserving populations were included in the included studies of the review can enable appropriate further analysis, highlight evidence gaps, and give insight into how applicable the findings of the studies, and therefore, the review findings, are to equity-deserving populations. The PRISMA-E checklist prompts review authors to decide a priori which populations will be examined and any subgroup analysis, including equity-deserving groups, will be conducted.^8^ In a review in which equity is not the primary focus, it will be important to consider the possible implications of the intervention process for disadvantaged groups, which will inform decisions about appropriate subgroup analysis, discussed in the analysis section. The PRO-EDI characteristics of included participants table has been designed to help review authors report participant information to facilitate consideration of equity and inclusion.^26^ Information about the participation prevalence ratio of populations in studies can indicate under-representation of equity-deserving groups, for example, dividing the percentage of female sepsis participants in studies by the percentage of females in the overall sepsis population.^27^ The lack of quality in conducting and reporting primary studies continues to be an issue^28^ and is problematic for reviews examining equity where detailed information about participants is crucial. When exploring equity, the review should consider the study sampling frames, participation rates and exclusion criteria for both the primary studies and the review itself.

### Representation of equity-deserving populations in the intervention—reach of interventions

Depending on the review question, it may be that multiple interventions are relevant, and different interventions could have different intended populations. Examining the reach of the interventions included in the review will compare the representativeness of the actual recipients of an intervention to the intended populations, see RE-AIM.^29^ As previously mentioned, pragmatic or real-world studies (including realist and pragmatic randomised controlled trials), rather than many randomised controlled trials, may be more likely to involve a broad range of the population. For example, primary studies using large administrative datasets may have the capacity to investigate equity if the datasets include relevant disadvantaged populations in sufficient numbers to allow robust analysis of these groups. However, within randomised trials, information about who is screened as being ineligible to participate and consent rates could be informative too.^30^ Collating information about the external validity of studies and reach of the interventions provides information about whether the review can synthesise information, such as acceptability of the intervention and delivery to different equity-deserving population groups.

## Re-examining the theory of change in relation to mechanisms of the intervention design and implementation

Investigating mechanisms is important at the analysis stage as well as at the planning stage of the review.^9^ It will be important to return to the theory of change or logic model^31^ to help articulate what differences between population groups are likely to be important, and exploring points in the logic model where inequity may exist. It may be useful to explore how mechanisms are activated differentially by population groups, that is, how mechanisms operate differently—or different mechanisms operate—between the intervention components and the intervention outcomes according to population group. This can include investigating how design components and how the implementation of an intervention impact different populations.

Re-examining the theory of change may involve revising the theory to incorporate findings from the review synthesis. For example, subgroup analysis might indicate that, while there were overall positive health outcomes, the intervention resulted in a tendency towards negative health outcomes for an equity-deserving population, indicating that there was a different pathway occurring for that equity-deserving group, requiring further investigation. This can be illustrated by smoking cessation mass media marketing campaigns, where more advantaged populations tend to be better able to engage with the campaign and change their behaviours.^5^ Re-examining the theory of change can be done by mechanistic reasoning, that is, examining inferences from mechanisms that may help further develop the theory of change. This requires demonstrating explicit links between the points of the theory of change, that is, the mechanisms, with evidence for those links while accounting for the complex nature of the mechanisms.^32^ A ‘metaframework’ has been developed to help build a theory in the review of what, how and why interventions work for different populations.^33^ Theories of how an intervention can lead to differential effects are examined in relation to the intervention components, implementation and context, and examining the strengths and limitations of those theories.^33^ Examining mechanisms may involve qualitative evidence to fill gaps in knowledge not provided by the quantitative data. Qualitative evidence will be valuable in understanding the perspectives of equity-deserving groups, how they experience the intervention and positive and negative impacts of the intervention.

## Conducting analysis for investigating equity of effectiveness

As much as possible, decisions about appropriate analysis to examine equity should be made when planning the review, in conjunction with considerations of relevant theory of change and mechanisms. Approaches include presenting inequity population results in separate tables, or rows of a table, to visually display any differences.^9^ Sometimes primary studies do not provide data that are amenable to meta-analysis; however, there are alternative synthesis methods that can synthesise data from the included studies when a meta-analysis is not feasible (eg, descriptive statistics or examining direction of effect through the use of structured tables, forest plots, harvest plots or effect direction plots).^34 35^ It is common to consider equity by conducting subgroup analysis, metaregression or both^9^; however, this is often not as simple as it may seem, and care is needed to ensure that any effects found are credible.^36^ Another approach, if sufficient appropriate data are available, is to conduct individual participant data meta-analysis, using the individual data from multiple studies rather than aggregate data.^37^

**Figure 1:**
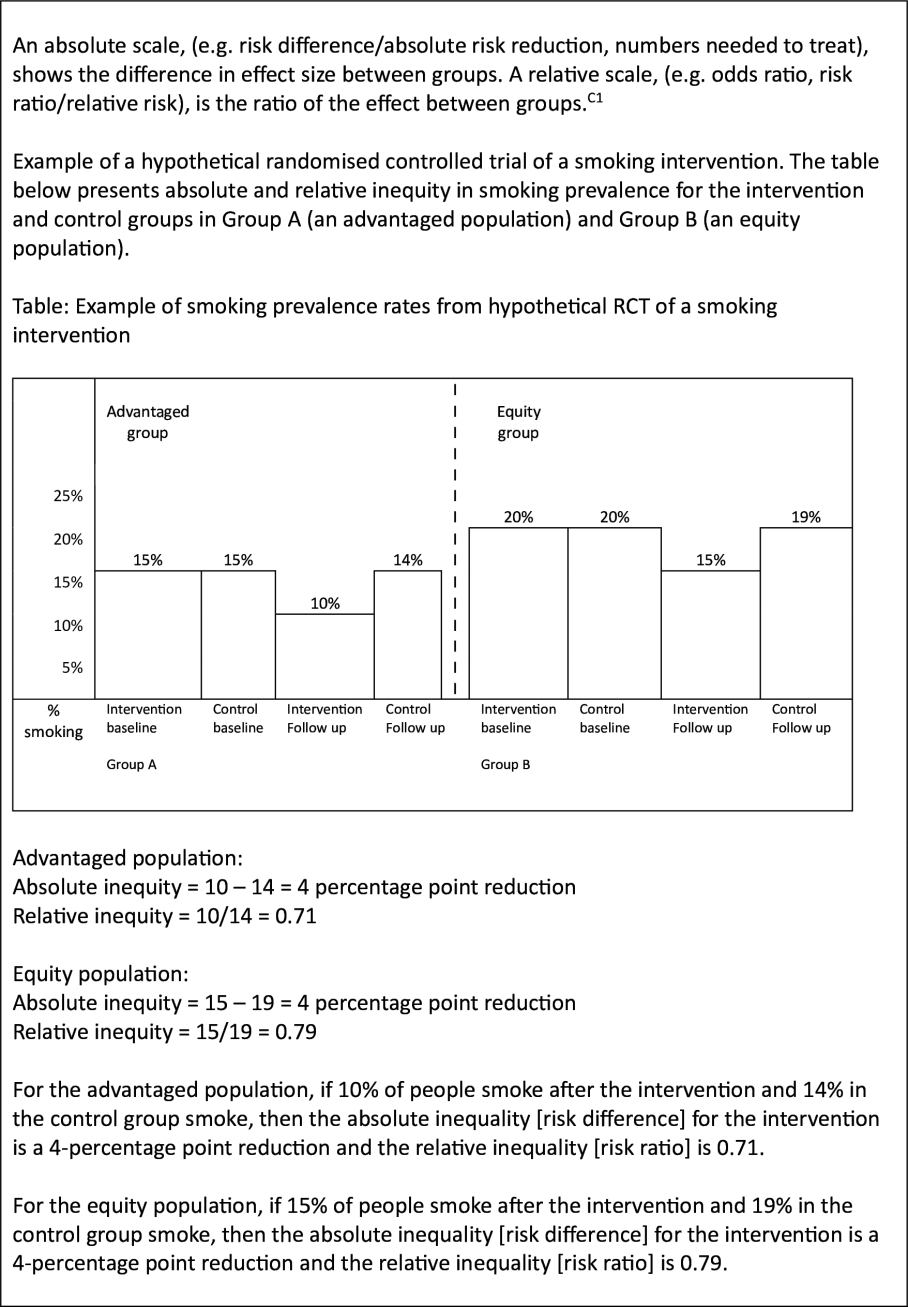
Example of absolute versus relative scales.

For subgroup analysis not to produce spurious results, it is preferable that a small number of prespecified hypotheses, backed by theory or indirect evidence, are examined.^38^ If it is appropriate to conduct subgroup analysis, conclusions about differences found in subgroup analysis will depend on the scale used; see explanation and example in figure 1. An intervention could be found to reduce health inequalities on an absolute scale, but analysis on a relative scale could produce different results (eg, the intervention increases health inequalities), or alternatively, the opposite may apply. It is, therefore, recommended that both absolute and relative risks are assessed,^8 9 39^ prespecification and justification are provided for the scale(s) used, particularly if using only one type of risk scale,^40^ and the scales are clearly reported.^8 9^

The statistical methods used have implications for interpreting results. Linear regression models typically used for continuous outcome variables are on an additive scale, whereas logistic, Poisson and Cox regression models typically used for dichotomous or time-to-event outcome measurements are on a multiplicative scale.^19^ This means that the default effect measures reported typically reflect these scales.^19^ However, it is possible to report results on alternative scales. For example, by creating a composite variable with a shared reference group of not receiving the intervention and having low social disadvantage, which is compared with other combinations of groups.

It is recommended that subgroup analysis is considered in combination with information from theories that explain or support any findings.^38 41^ Reporting analysis results should indicate clearly whether the analysis was planned and in the review protocol or decided on during the analysis stage.^8^

## Economic evidence and equity

Incorporating economic evidence into evidence synthesis can be either a full systematic review of the economic evidence or a ‘brief economic commentary’.^42^ Assessing the cost-effectiveness of an intervention is a necessary consideration for policymakers deciding how to spend finite funds, but is usually limited to focusing on efficiency and overall average health outcomes.^43^ Therefore, a review examining equity requires exploring evidence that can better incorporate equity, such as extended effectiveness analysis^44^ or distributional cost-effectiveness analysis.^45^

Distributional cost-effectiveness analysis takes account of equity when assessing the costs of interventions and can transparently incorporate social value judgements about inequitable health variation into the analysis. This can provide policy decision makers and interest holders with various analysis results, depending on different social value judgements made, which can differ across societies and communities.^45^ These methods aim to understand the tradeoff between maximising equity and maximising population health. Examining distributional cost-effectiveness may discover that an intervention is more, or less, cost-effective for certain populations. This could have implications for policymaking decisions; higher costs of an intervention may be acceptable if the intervention is shown to reduce health inequity. Conducting distributional cost-effectiveness analysis requires careful consideration of whether adequate data are available, for example, sociodemographic data for the equity-deserving populations.^46^

## Interpreting results in relation to equity

Interpreting results in relation to equity involves considering results of statistical synthesis and any subgroup analysis in relation to the theory of change, the external validity of the included studies and reach of the interventions and evidence from additional sources.^8^ It should be clear how cautiously results should be treated.^9^ It may be appropriate to highlight any new theory or evidence that has been found, which may be areas for further research.^8 9^

Approaches that may help interpret results include producing tables or figures, such as harvest plots,^47^ which show results ordered by subpopulation characteristics, intervention components and/or context. Other approaches, such as intervention component analysis^48^ and qualitative comparative analysis,^49^ may be helpful for examining equity. Intervention component analysis identifies key features of an intervention and barriers and facilitators to its implementation.^48^ Qualitative comparative analysis gathers and compares in-depth knowledge of studies using set theory to explore the causal contribution of different conditions, such as intervention components and context.^49^ While not all of these examples will be possible in every review, our intention is to provide options to inspire review authors to explore methods that are suitable for their particular review.

Guidance for assessing the certainty, that is, the quality and confidence, of evidence for an intervention is provided by the Grading Recommendations Assessment and Development Evidence (GRADE) guideline for equity.^50^ To ensure that this assessment captures any inequities, it is recommended that health equity characteristic(s) are included as an outcome, determining the importance of those characteristics from evidence and input from relevant interest holders of equity-deserving groups. The guideline additionally recommends that differences in treatment effects are examined on both relative and absolute scales (see above) and how directly the population groups of the relevant studies match the equity-deserving population(s) of interest is assessed.^50^

## Conclusion

This article provides key points for incorporating equity into all systematic reviews. The focus of the review will have different implications depending on whether the review is focused on an inequity issue or a broader review assessing equity as a secondary consideration. It is important that the review considers the perspectives of equity-deserving groups and organisations, and we note the need for guidance on intersectionality. Making conclusions about equity involves bringing together results from across different sections of the review. However, not all the methods discussed here will be possible in every review. Our intention is to provide options for authors to consider for their own systematic review. While incorporating equity into reviews relates to often-identified problems of primary studies not clearly reporting populations or interventions and not conducting research on equity-deserving populations, this article aims to help authors of systematic reviews make best use of the evidence that is available.

## Supplementary material

10.1136/jech-2025-224306 online supplemental file 1

## Data Availability

No data are available.
