# Supplementary material for: Incorporating an equity perspective in systematic reviews of interventions: potential methodological approaches
Source: J Epidemiol Community Health. 2025 Dec 19;80(5):e224306. doi: 10.1136/jech-2025-224306 (PMC13151474; doi:10.1136/jech-2025-224306)
Supplement: online supplemental file 1 [file jech-80-5-s001.pdf]

Incorporating an equity perspective in systematic reviews of interventions: potential methodological approaches – supplementary file.

- **References for Table 1**
- **References for Table 2**
- **References for Table 3**
- **References for Figure 1**

References for Table 1 Terminology related to health equity

A1. WHO Commission on Social Determinants of Health. Closing the gap in a generation: health equity through action on the social determinants of health: Commission on Social Determinants of Health final report. Geneva: World Health Organization 2008.

A2. Oickle D, Clement C. Glossary of health equity concepts for public health action in the Canadian context. Journal of Epidemiology and Community Health 2019;73(9):802-05. doi: 10.1136/jech-2018-210851

A3. Dewidar O, Pardo JP, Welch V, et al. Operationalizing the GRADE-equity criterion to inform guideline recommendations: application to a medical cannabis guideline. Journal of Clinical Epidemiology 2024;165:111185. doi: https://doi.org/10.1016/j.jclinepi.2023.10.001

A4. Akl EA, Khabsa J, Petkovic J, et al. “Interest-holders”: A new term to replace “stakeholders” in the context of health research and policy. Cochrane Evidence Synthesis and Methods 2024;2(11):e70007. doi: https://doi.org/10.1002/cesm.70007

A5. O'Neill J, Tabish H, Welch V, et al. Applying an equity lens to interventions: using PROGRESS ensures consideration of socially stratifying factors to illuminate inequities in health. Journal of Clinical Epidemiology 2014;67(1):56-64.

A6. McCann L, Johnson L, Gkiouleka A, et al. A Novel Framework for Equity-Focused Evidence Synthesis: EQUALS MAP. Available at SSRN 4703772

References for Table 2 Guidance for researchers incorporating equity into systematic reviews

B1. Welch VA, Petkovic J, Jull J, et al. Chapter 16: Equity and specific populations. Cochrane Handbook for Systematic Reviews of Interventions version 64, 2023.

B2. Welch V, Petticrew M, Petkovic J, et al. Extending the PRISMA statement to equity-focused systematic reviews (PRISMA-E 2012): explanation and elaboration. *Journal of Clinical Epidemiology* 2016;70:68-89.

B3. Campbell and Cochrane Equity Methods Group 2025 [Available from: https://methods.cochrane.org/equity/.]

B4. Welch VA, Akl EA, Guyatt G, et al. GRADE equity guidelines 1: considering health equity in GRADE guideline development: introduction and rationale. *Journal of Clinical Epidemiology* 2017;90:59-67. doi: 10.1016/j.jclinepi.2017.01.014

B5. Akl EA, Welch V, Pottie K, et al. GRADE equity guidelines 2: considering health equity in GRADE guideline development: equity extension of the guideline development checklist. *Journal of Clinical Epidemiology* 2017;90:68-75. doi: 10.1016/j.jclinepi.2017.01.017

B6. Welch VA, Akl EA, Pottie K, et al. GRADE equity guidelines 3: considering health equity in GRADE guideline development: rating the certainty of synthesized evidence. *Journal of Clinical Epidemiology* 2017;90:76-83.

B7. Pottie K, Welch V, Morton R, et al. GRADE equity guidelines 4: considering health equity in GRADE guideline development: evidence to decision process. *Journal of Clinical Epidemiology* 2017;90:84-91. doi: 10.1016/j.jclinepi.2017.08.001

B8. Aluko P, Graybill E, Craig D, et al. Chapter 20: Economic evidence. In: Higgins JPT TJ, Chandler J, Cumpston M, Li T, Page MJ, Welch VA (editors), ed. Cochrane Handbook for Systematic Reviews of Interventions version 64 (updated August 2023): Cochrane, 2023.

B9. O'Neill J, Tabish H, Welch V, et al. Applying an equity lens to interventions: using PROGRESS ensures consideration of socially stratifying factors to illuminate inequities in health. *Journal of Clinical Epidemiology* 2014;67(1):56-64.

B10. Rehfuess EA, Stratil JM, Scheel IB, et al. The WHO-INTEGRATE evidence to decision framework version 1.0: integrating WHO norms and values and a complexity perspective. *BMJ Global Health* 2019;4(Suppl 1):e000844. doi: 10.1136/bmjgh-2018-000844

B11. Heidari S, Babor TF, De Castro P, et al. Sex and Gender Equity in Research: rationale for the SAGER guidelines and recommended use. *Research Integrity and Peer Review* 2016;1(1):2. doi: 10.1186/s41073-016-0007-6

B12. Treweek S, Bruhn H. PRO EDI participant characteristics table 22/3/2024 2024 [Available from: https://www.trialforge.org/trial-diversity/pro-edi-improving-how-equity-diversity-and-inclusion-is-handled-in-evidence-synthesis/ accessed 12 Aug 2024.

B13. Centre for Addiction and Mental Health (CAMH). Health Equity Impact Assessment (HEIA) 2019 [Available from: https://www.porticonetwork.ca/web/heia/home.]

B14. World Health Organization (WHO). Health Equity Assessment Toolkit (HEAT): Software for exploring and comparing health inequalities in countries. Geneva, 2024.

References for Table 3

T1. Wagner C, Griesel M, Mikolajewska A, et al. Systemic corticosteroids for the treatment of COVID-19: Equity-related analyses and update on evidence. *Cochrane Database Syst Rev* 2022;11(11):Cd014963. doi: 10.1002/14651858.CD014963.pub2 [published Online First: 2022/11/18]

T2. Coren E, Hossain R, Pardo Pardo J, et al. Interventions for promoting reintegration and reducing harmful behaviour and lifestyles in street - connected children and young people. *Cochrane Database of Systematic Reviews* 2016(1) doi: 10.1002/14651858.CD009823.pub3

T3. Aggarwal S, Wright J, Morgan A, et al. Religiosity and spirituality in the prevention and management of depression and anxiety in young people: a systematic review and meta-analysis. BMC Psychiatry 2023;23(1):729. doi: 10.1186/s12888-023-05091-2

T4. Attwood S, van Sluijs E, Sutton S. Exploring equity in primary-care-based physical activity interventions using PROGRESS-Plus: a systematic review and evidence synthesis. *International Journal of Behavioral Nutrition and Physical Activity* 2016;13(1):60. doi: 10.1186/s12966-016-0384-8

T5. Valdebenito S, Gaffney H, Arosemena-Burbano MJ, et al. School-Based Interventions for Reducing Disciplinary School Exclusion. An Updated Systematic Review. Campbell Systematic Reviews 2025;21(4):e70063

T6. Brown T, Platt S, Amos A. Equity impact of population-level interventions and policies to reduce smoking in adults: a systematic review. *Drug Alcohol Depend* 2014;138:7-16. doi: 10.1016/j.drugalcdep.2014.03.001 [published Online First: 2014/03/29]

T7. Coll-Planas L, Nyqvist F, Puig T, et al. Social capital interventions targeting older people and their impact on health: a systematic review. *Journal of Epidemiology and Community Health* 2017;71(7):663-72. doi: 10.1136/jech-2016-208131

T8. Gardner F, Leijten P, Harris V, et al. Equity effects of parenting interventions for child conduct problems: a pan-European individual participant data meta-analysis. *The Lancet Psychiatry* 2019;6(6):518-27. doi: 10.1016/S2215-0366(19)30162-2

T9. Tanner et al. inequalities in common mental health disorders? A protocol for a systematic review of quantitative and qualitative studies. *International Journal of Environmental Research and Public Health* 2021;18(24):12978.

T10. Adegbosin AE, Zhou H, Wang S, et al. Systematic review and meta-analysis of the association between dimensions of inequality and a selection of indicators of Reproductive, Maternal, Newborn and Child Health (RMNCH). *J Glob Health* 2019;9(1):010429. doi: 10.7189/jogh.09.010429 [published Online First: 2019/05/28]

T11. Spencer JC, Spees LP, Biddell CB, et al. Inclusion of marginalized populations in HPV vaccine modeling: A systematic review. *Preventive Medicine* 2024;182:107941.

T12. Saad A, Magwood O, Aubry T, et al. Mobile interventions targeting common mental disorders among pregnant and postpartum women: An equity-focused systematic review. *PLOS ONE* 2021;16(10):e0259474. doi: 10.1371/journal.pone.0259474

T13. Prady SL, Endacott C, Dickerson J, et al. Inequalities in the identification and management of common mental disorders in the perinatal period: An equity focused re-analysis of a systematic review. *PLoS One* 2021;16(3):e0248631. doi: 10.1371/journal.pone.0248631 [published Online First: 2021/03/16]

T14. Wang M, Jiang S, Li B, et al. Synthesized economic evidence on the cost-effectiveness of screening familial hypercholesterolemia. *Global Health Research and Policy* 2024;9(1):38. doi: 10.1186/s41256-024-00382-x

T15. Colclough Z, Estrella MJ, Joyce JM, et al. Equity considerations in clinical practice guidelines for traumatic brain injury and the criminal justice system: A systematic review. *PLOS Medicine* 2024;21(8):e1004418. doi: 10.1371/journal.pmed.1004418

Reference for Figure 1

C1. Andersen LW. Absolute vs. relative effects—implications for subgroup analyses. *Trials* 2021;22(1):50. doi: 10.1186/s13063-020-05005-7
